# Acute kidney disease in hospitalized acute kidney injury patients

**DOI:** 10.7717/peerj.11400

**Published:** 2021-05-24

**Authors:** Ping Yan, Xiang-Jie Duan, Yu Liu, Xi Wu, Ning-Ya Zhang, Fang Yuan, Hao Tang, Qian Liu, Ying-Hao Deng, Hong-Shen Wang, Mei Wang, Shao-bin Duan

**Affiliations:** 1 Department of Nephrology, The Second Xiangya Hospital of Central South University; Hunan Key Laboratory of Kidney Disease and Blood Purification, Changsha, Hunan, China; 2 Information Center, The Second Xiangya Hospital of Central South University, Changsha, Hunan, China; 3 Nutrition and Exercise Physiology, Teachers College, Columbia University, New York, United States of America

**Keywords:** Acute kidney injury, Kidney disease, Mortality, Renal dysfunction, Outcome

## Abstract

**Background:**

Acute kidney injury (AKI) and chronic kidney disease (CKD) have become worldwide public health problems, but little information is known about the epidemiology of acute kidney disease (AKD)—a state in between AKI and CKD. We aimed to explore the incidence and outcomes of hospitalized patients with AKD after AKI, and investigate the prognostic value of AKD in predicting 30-day and one-year adverse outcomes.

**Methods:**

A total of 2,556 hospitalized AKI patients were identified from three tertiary hospitals in China in 2015 and followed up for one year.****AKD and AKD stage were defined according to the consensus report of the Acute Disease Quality Initiative 16 workgroup. Multivariable regression analyses adjusted for confounding variables were used to examine the association of AKD with adverse outcomes.

**Results:**

AKD occurred in 45.4% (1161/2556) of all AKI patients, 14.5% (141/971) of AKI stage 1 patients, 44.6% (308/691) of AKI stage 2 patients and 79.6% (712/894) of AKI stage 3 patients. AKD stage 1 conferred a greater risk of Major Adverse Kidney Events within 30 days (MAKE30) (odds ratio [OR], 2.36; 95% confidence interval 95% CI [1.66–3.36]) than AKD stage 0 but the association only maintained in AKI stage 3 when patients were stratified by AKI stage. However, compared with AKD stage 0, AKD stage 2–3 was associated with higher risks of both MAKE30 and one-year chronic dialysis and mortality independent of the effects of AKI stage with OR being 31.35 (95% CI [23.42–41.98]) and 2.68 (95% CI [2.07–3.48]) respectively. The association between AKD stage and adverse outcomes in 30 days and one year was not significantly changed in critically ill and non-critically ill AKI patients. The results indicated that AKD is common among hospitalized AKI patients. AKD stage 2–3 provides additional information in predicting 30-day and one-year adverse outcomes over AKI stage. Enhanced follow-up of renal function of these patients may be warranted.

## Introduction

Acute kidney injury (AKI) is a common disorder worldwide which affects 7–18% of hospital inpatients and 30–70% of critically ill patients (Lewington, Cerda & Mehta, 2013). The disorder is associated with considerable morbidity, mortality and high costs and brings great economic burden to the family and society (Hoste et al., 2018). An episode of AKI, even mild AKI can increase the risk of chronic kidney disease (CKD), end stage renal disease and premature death (Lameire et al., 2013; Mehta et al., 2015). Increased severity of AKI is also associated with a greater risk for death (Coca et al., 2009; Susantitaphong et al., 2013). However, few effective methods or therapies are available to reverse AKI in clinical practice (Kidney Disease: Improving Global Outcomes KDIGO, 2012; Basile et al., 2016; Yang et al., 2016). Herein, understanding the mechanism and clinical features of renal progression/recovery after an episode of AKI has gained great attention from researchers (Goldstein et al., 2014; He et al., 2017; Liu et al., 2019).

Recently, acute kidney disease (AKD) has been a rising concern. AKD is defined as persistent renal damage and/or renal dysfunction for a duration of 7 to 90 days after exposure to an AKI initiating event by the Acute Disease Quality Initiative (ADQI) 16 Workgroup (Chawla et al., 2017). It is proposed to define the course of disease after AKI and represents a subpopulation whose pathophysiological processes are ongoing. AKI and AKD reflect renal function status in different time periods during the disease process. Although studies have shown that AKD is associated with increased risks of mortality and renal function decline after hospital discharge (Hsu et al., 2020; James et al., 2019; Kofman et al., 2019; Matsuura et al., 2020; Mizuguchi et al., 2018), few targeted on patients with AKD after AKI, and the epidemiology of hospitalized patients with AKD after AKI is largely unknown. Whether AKD acts as an important intermediate stage for progression to renal dysfunction, chronic dialysis and mortality after AKI remains to be found.

To address this issue, we conducted a multi-center retrospective study of hospitalized AKI patients in China. We aimed to investigate the incidence and outcomes of hospitalized patients with AKD after AKI and to examine whether AKD adds additional prognostic information over AKI stage in predicting 30-day and one-year adverse outcomes.

## Materials and Methods

### Study design, setting and population

**Figure 1:**
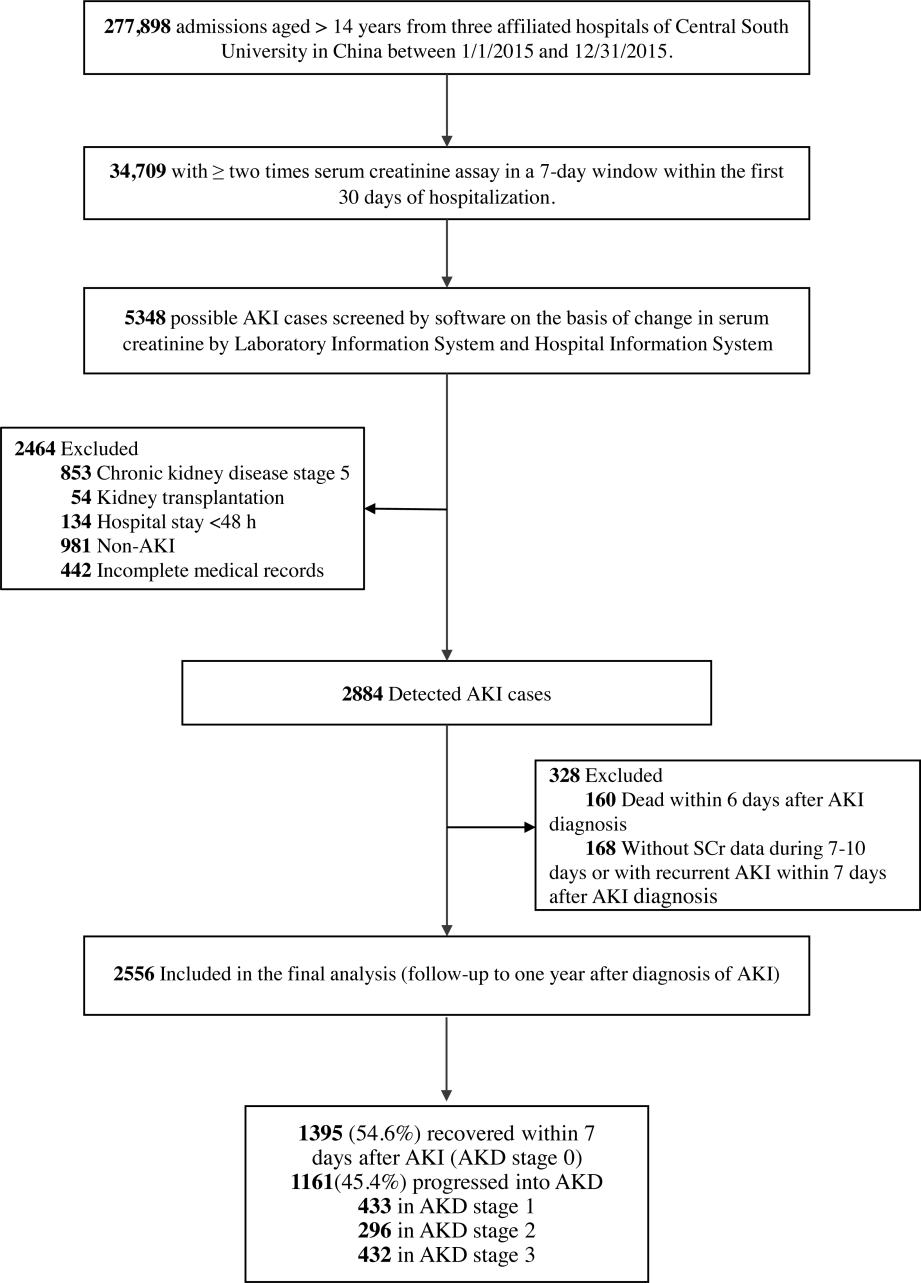
Study flow chart. AKI, acute kidney injury; AKD, acute kidney disease.

The multi-center retrospective cohort study identified AKI patients from hospitalized patients aged more than 14 years in three affiliated hospitals of Central South University in China from January 1st, 2015 to December 31st, 2015. Study procedures were shown in Fig. 1. AKI was determined according to the 2012 Kidney Disease: Improving Global Outcomes Clinical Practice Guideline for Acute Kidney Injury (Kidney Disease: Improving Global Outcomes KDIGO, 2012) by the serum creatinine (SCr) level. Increase in SCr by ≥ 0.3 mg/dl within 48 h or increase in SCr to ≥ 1.5 times baseline within 7 days was used to identify AKI patients. Patients with CKD stage 5 (admission diagnosis including CKD stage 5, end stage renal disease or uremia), kidney transplantation (admission diagnosis including kidney transplantation or kidney transplant status), hospital stay <48 h, non-AKI or incomplete medical records were excluded. For patients with multiple hospitalizations, only the first hospitalization was included in the analysis. The Medical Ethics Committee of the Second Xiangya Hospital of Central South University approved the study protocol (2013-S061) and waived the patient consent. This project has been registered in Chinese Clinical Trial Registry (ChiCTR 1800019857).

### Identification of AKD

AKD was defined as a condition in which AKI stage 1 or greater persists for more than 6 days and less than 90 days after an AKI initiating event according to the consensus report of the ADQI 16 Workgroup (Chawla et al., 2017). AKD stage 1 was defined as SCr being 1.5–1.9 times of the baseline level, AKD stage 2 as SCr being 2.0–2.9 times of the baseline level, and AKD stage 3 as SCr being ≥3.0 times of the baseline level or ongoing need for renal replacement therapy. Patients with SCr <1.5 times of baseline level were categorized as AKD stage 0 (non-AKD group). AKD and AKD stage were identified based on the SCr level on the 7th day or the day nearest to the 7th day but not more than the 10th day after the diagnosis of AKI. Baseline SCr was defined as the mean SCr value that was available between 7-365 days prior to admission (Siew et al., 2012). For patients without a reliable baseline SCr before admission and with no evidence of baseline CKD, a back-estimation of the baseline SCr was performed using the Modification of Diet in Renal Disease study equation assuming that the baseline eGFR is 75 ml/min per 1.73 m^2^ (Bellomo et al., 2004).

### Data collection and definition

Patients’ information was extracted from Hospital Information System, Laboratory Information System and medical records, which included age, sex, clinical departments, comorbidities, diagnosis on admission and discharge, surgeries, invasive procedures, all-cause in-hospital death, as well as laboratory test results and time. Identification and classification of AKI (2012), AKI stage, AKI type, comorbidity, Charlson comorbidity index (CCI) (Charlson et al., 1994), organ failure (Blanco et al., 2008) and sepsis (Bone et al., 1992) were evaluated by trained nephrologists through reviewing medical records and laboratory data. AKI stage was defined according to the 2012 KDIGO guide for AKI ( Kidney Disease: Improving Global Outcomes KDIGO, 2012) and the highest stage within the first 6 days of AKI diagnosis was used to determine the AKI stage. Community-acquired AKI was identified when patients met the KDIGO AKI definition according to SCr change on the first day of admission; hospital-acquired AKI was identified when patients who developed AKI did not meet community-acquired AKI criteria. Critically ill patients were identified as those who were admitted to intensive care units when AKI was diagnosed. Patients who could not be classified as critically ill patients were regarded as non-critically ill patients. Presence of comorbidities was determined by the diagnosis codes at admission and discharge. The burden of comorbidities was evaluated by CCI. Organ failure (cardiovascular system dysfunction, the arterial systolic blood pressure ≤ 90 mm Hg or mean arterial pressure ≤ 70 mm Hg or use of vasopressors; kidney dysfunction, urine output <0.5 ml/kg /h despite adequate blood volume, or creatinine >1.9 mg/dl; respiratory system dysfunction, hypoxemia with PaO2 <60 mm Hg or mechanical ventilation; hematologic dysfunction, the platelet count <80,000/mm^3^; liver dysfunction, bilirubin ≥ 3 mg/dl; nervous system dysfunction, use of sedative), and diagnosis of anemia (hemoglobin ≤ 10 g/dl), hypoalbuminemia (serum albumin <30 g/l), proteinuria (dipstick urinalysis protein positive), and hyperuricemia (serum uric acid ≥7.0 mg/dl in men and 6.0 mg/dl in women) were determined by the data within seven days prior to AKD diagnosis. The worst result was used if there were multiple results for the same test. Data on hospital operation were determined by patients’ medical orders information and collected within three days prior to AKD diagnosis.

### Endpoints and follow-up

The primary endpoint was the proportion of patients who met one or more criteria for Major Adverse Kidney Events within 30 days (MAKE30) (Kellum, Zarbock & Nadim, 2017; Semler et al., 2016) after diagnosis of AKI which consists of all-cause mortality (hereafter referred to as mortality), new receipt of renal replacement therapy, or persistent renal dysfunction (defined as the creatinine value ≥ 200% of the baseline value). All events were censored at hospital discharge or 30 days after the diagnosis of AKI, whichever came first. The second endpoint was the recipient of chronic dialysis or mortality one year after the diagnosis of AKI. MAKE30 and chronic dialysis in one year were identified through reviewing all relevant medical records (Hospital Information System, Laboratory Information System and out-patients records), making phone calls and sending messages. Dialysis performed during hospitalization was determined based on patients’ medical orders information which included ‘hemodialysis’ or ‘peritoneal dialysis’. Chronic dialysis was further determined by referring to the Chinese National Renal Data System. Death was confirmed through data linkage to the Chinese Center for Disease Control and Prevention cause-of-death reporting system which is a national administrative registry responsible for collection and management of death information from all provinces in China.

### Statistical analysis

Patients’ baseline characteristics and outcomes were compared between AKD stages using One-way ANOVA test or Kruskal-Wallis test for continuous variables and chi-square test for categorical variables as appropriate. Multiple comparisons between AKD stages were made by Student-Newman-Keuls test for continuous variables and Scheffe’s confidence interval method for categorical variables as appropriate. Multivariable logistic regression analysis was used to estimate the impact of AKD stage on persistent renal dysfunction and new receipt of renal replacement therapy in 30 days, MAKE30, one-year chronic dialysis as well as composite adverse outcomes in one year. Cox proportional hazard regression model was used to estimate the hazard ratio of AKD on 30-day and one-year mortality. Variables that were considered clinically relevant or associated with outcomes were adjusted in the multivariable models. Proportional hazards assumption was assessed by the curves of log[-logS(t)] to t test for each covariable. Multivariable logistic regression analysis was used to determine the predictors of AKD stage 2–3 and establish the risk prediction model. Baseline variables that were considered clinically relevant or that showed a univariable relationship with outcome were entered into the model and selected by the forward selection method. Discrimination and calibration of the model were tested by area under the receiver operating characteristic curve (AUROC) and Hosmer and Lemeshow test.

Statistical analyses were performed using SPSS 18.0 (IBM Corporation). *P* < 0.05 was considered statistically significant.

## Results

### Incidence of AKD

The overall incidences of AKI and AKD in hospitalized patients were 8.31% (2884/34709) and 3.34% (1161/34709) respectively. 2,556 AKI patients meeting the inclusion criteria were enrolled in our analysis. AKD occurred in 45.4% (1161/2556) of all AKI patients, 14.5% (141/971) of AKI stage 1 patients, 44.6% (308/691) of AKI stage 2 patients and 79.6% (712/894) of AKI stage 3 patients. Characteristics of the study population stratified by AKD stages were presented in Table 1. The prevalences of AKD stage 1 and stage 2–3 were 16.9% and 28.5% respectively. AKD stage 2–3 tended to occur in patients who were older and accompanied with more comorbidities. A larger percentage of patients with AKI stage 3, intrinsic renal AKI as well as mechanical ventilation were also observed in AKD stage 2–3.

### Outcomes of AKD patients

**Figure 2:**
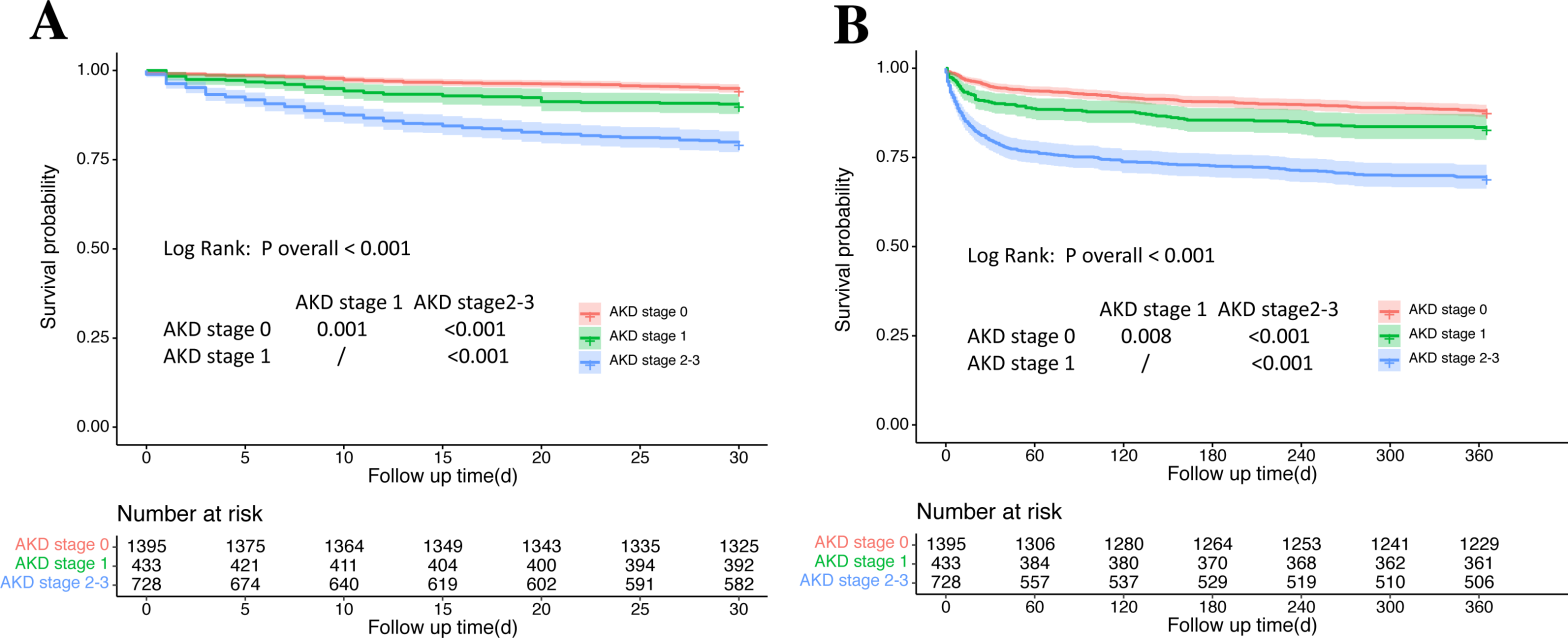
Kaplan–Meier survival curves for 30-day and one-year mortality among AKD stages. (A) Kaplan–Meier survival curves for 30-day mortality among AKD stages. (B) Kaplan–Meier survival curves for one-year mortality among AKD stages. AKD, acute kidney disease.

Outcomes of patients stratified by AKD stage were provided in Table 2. Kaplan–Meier survival curves for 30-day and one-year mortality among AKD stages were shown in Fig. 2. During the one-year follow-up, 26.8% (684/2556) of patients developed the composite endpoints of MAKE30, of whom 13.5% (92/684) came from the AKD stage 0 group, 11.0% (75/684) from the AKD stage 1 group and 75.6% (517/684) from the AKD stage 2–3 group. 19.0% (486/2556) of patients developed the composite endpoints of adverse outcomes (chronic dialysis and mortality) in one year, with 35.2% (171/486) from the AKD stage 0 group, 15.0% (73/486) from the AKD stage 1 group and 49.8% (242/486) from the AKD stage 2–3 group. A stepwise increase in the incidence of MAKE30 and one-year adverse outcomes was observed as the AKD stage got higher. Compared with AKD stage 0 and 1, patients with AKD stage 2–3 consistently showed significantly higher incidences of developing all adverse outcomes in 30 days and one year. AKD stage 1 only had higher incidences in persistent renal dysfunction and mortality in 30 days than AKD stage 0 (Table 2).

Association between AKD stage and 30-day as well as one-year adverse outcomes was shown in Table 3. Fully adjusted multivariable regression analysis indicated that AKD stage 1 mainly showed an increased risk of persistent renal function in 30 days, while AKD stage 2–3 conferred remarkably higher risks in receiving renal replacement therapy, developing persistent renal dysfunction and mortality in 30 days along with chronic dialysis and mortality in one year. OR/HR for covariables adjusted in the multivariable regression model were shown in Tables S1–S7.

**Table 1:** Characteristics of study population stratified by AKD stages.

Variables	AKD stage 0 *n* = 1395 (54.6%)	AKD stage 1 *n* = 433 (16.9%)	AKD stage 2–3 *n* = 728 (28.5%)	*P* value^*^
Age(years)	53.3 ± 16.0	54.4 ± 17.0	56.0 ± 16.9^a^	0.002
Age group,				<0.001
15–64 years	1033 (74.1%)	300 (69.3%)	480 (65.9%)^a^	
≥ 65 years	362 (25.9%)	133 (30.7%)	248 (34.1%)^a^	
Male	847 (60.7%)	294 (67.9%)^a^	462 (63.5%)	0.023
ICU admission	437 (31.3%)	130 (30.0%)	254 (34.9%)	0.147
AKI stage^b^^,^^c^				<0.001
1	830 (59.5%)	102 (23.6%)	39 (5.4%)	
2	383 (27.5%)	148 (34.2%)	160 (22.0%)	
3	182 (13.0%)	183 (42.3%)	529 (72.7%)	
AKI type				<0.001
CA-AKI	525 (37.6%)	239 (55.2%)^a^	345 (47.4%)^a^^,^^d^	
HA-AKI	870 (62.4%)	194 (44.8%)^a^	383 (52.6%)^a^^,^^d^	
AKI classification^b^				<0.001
Pre-renal	996 (71.4%)	250 (57.7%)	416 (57.1%)	
Intrinsic-renal	213 (15.3%)	124 (28.6%)	224 (30.8%)	
Post-renal	85 (3.3%)	35 (8.1%)	50 (6.9%)	
Unclassified	101 (7.2%)	24 (5.5%)	38 (5.2%)	
Comorbidity				
Hypertension	449 (32.2%)	151 (34.9%)	284 (39.0%)^a^	0.007
Diabetes	251 (18.0%)	86 (19.9%)	148 (20.3%)	0.374
CKD	37 (2.7%)	33 (7.6%)^a^	34 (4.7%)	<0.001
Myocardial infarction	35 (2.5%)	22 (5.1%)	39 (5.4%)^b^	0.001
Congestive heart failure	197 (14.1%)	81 (18.7%)	144 (19.8%)^b^	0.002
Cerebrovascular disease	186 (13.3%)	56 (12.9%)	90 (12.4%)	0.819
Chronic liver disease	327 (23.4%)	107 (24.7%)	221 (30.4%)^a^	0.002
Cancer	402 (28.8%)	85 (19.6%)^a^	156 (21.4%)^a^	<0.001
Sepsis	97 (7.0%)	43 (9.9%)	108 (14.8%)^a^	<0.001
CCI (≥2)	764 (54.8%)	240 (55.4%)	448 (61.5%)^a^	0.009
Organ failure (≥2)	271 (19.4%)	131 (30.3%)^a^	310 (42.6%)^a^^,^^d^	<0.001
Laboratory data				
Anemia,	353 (25.3%)	150 (34.6%)^a^	293 (40.2%)^a^	<0.001
Hypoalbuminemia	283 (20.3%)	137 (31.6%)^a^	267 (36.7%)^a^	<0.001
Proteinuria	142 (10.3%)	92 (21.4%)^a^	165 (23.0%)^a^	<0.001
Hyperuricemia	459 (32.9%)	233 (53.8%)^a^	435 (59.8%)^a^	<0.001
Baseline SCr (µmol/L)	73.6 ± 24.9	89.6 ± 34.5^a^	85.2 ± 25.6^a^^,^^d^	<0.001
Hospital operation				
Cardiovascular Surgery	105 (7.5%)	26 (6.0%)	55 (7.6%)	0.535
Mechanical Ventilation	213 (15.3%)	93 (21.5%)^a^	210 (28.8%)^a^^,^^d^	<0.001
Hospital stay (d)	15 (9–22)	16 (10–26)^a^	15 (10–25)^a^	<0.001

**Notes.**

AKD acute kidney disease ICU intensive care unit AKI acute kidney injury CA-AKI community-acquired acute kidney injury HA-AKI hospital-acquired acute kidney injury CKD chronic kidney disease CCI Charlson comorbidity index SCr serum creatinine

* Comparison was made among AKD stage 0, 1 and 2–3.

a *p* < 0.05 compared with AKD stage 0.

b Overall *P* < 0.05 compared with AKD stage 0 in both AKD stage 1 and AKD stage 2–3.

c Overall *P* < 0.05 between AKD stage 1 and AKD stage 2–3.

d *p* < 0.05 compared with AKD stage 1.

**Table 2:** Outcomes of hospitalized AKI patients stratified by AKD stage.

Outcomes	All patients *N* = 2556 *n*(%)	AKD stage 0 *N* = 1395 *n*(%)	AKD stage 1 *N* = 433 *n*(%)	AKD stage 2–3 *N* = 728 *n*(%)	*P* value^*^
Major Adverse Kidney Events within 30 days					
PRD	561 (21.9%)	19 (1.4%)	40 (9.2%)^a^	502 (69.0%)^a^^,^^b^	<0.001
New RRT	66 (2.6%)	3 (0.2%)	6 (1.4%)	57 (7.8%)^a^^,^^b^	<0.001
Mortality	260 (10.2%)	72 (5.2%)	41 (9.5%)^a^	147 (20.2%)^a^^,^^b^	<0.001
Total	684 (26.8%)	92 (6.6%)	75 (17.3%)^a^	517 (71.0%)^a^^,^^b^	<0.001
One-year adverse outcomes					
Chronic dialysis	26 (1.1%)	4 (0.3%)	1 (0.2%)	21 (3.0%)^a^^,^^b^	<0.001
Mortality	461 (18.0%)	167 (12.0%)	72 (16.6%)	222 (30.5%)^a^^,^^b^	<0.001
Total	486 (19.0%)	171 (12.3%)	73 (16.9%)	242 (33.2%)^a^^,^^b^	<0.001

**Notes.**

AKI acute kidney injury AKD acute kidney disease PRD persistent renal dysfunction RRT renal replacement therapy

* Comparison was made among AKD stage 0, 1 and 2–3.

a *P* < 0.05 compared with AKD stage 0.

b *P* < 0.05 compared with AKD stage 1.

### Association between AKI stage, AKD stage and adverse outcomes in 30 days and one year

**Figure 3:**
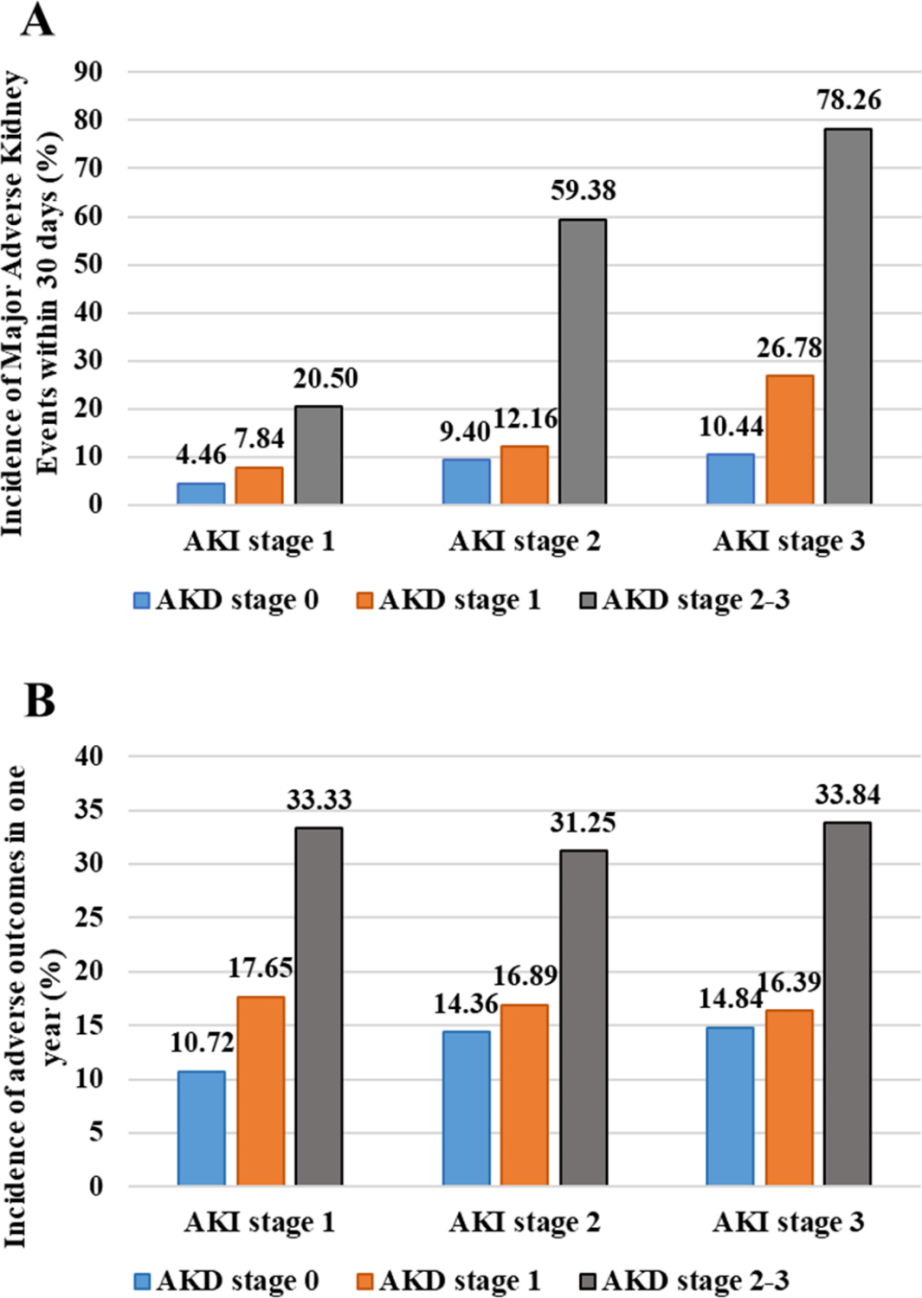
Incidences of Major Adverse Kidney Events within 30 days and one-year adverse outcomes in patients stratified by AKI stage and AKD stage. (A) Incidences of Major Adverse Kidney Events within 30 days; (B) Incidences of one-year adverse outcomes. AKI, acute kidney injury; AKD, acute kidney disease.

To rule out the impact of AKI stage on the association between AKD and 30-day as well as one-year adverse outcomes, the incidences of MAKE30 and one-year adverse outcomes in patients stratified by both AKI stage and AKD stage were analyzed and shown in Fig. 3. In each stratum of AKI, the incidences of MAKE30 and one-year adverse outcomes went up as AKD stage got higher and increased remarkably from AKD stage 1 to AKD stage 2–3. When stratified by AKD stage, the occurrence of MAKE30 increased with the increase of AKI stage while the occurrence of one-year adverse outcomes did not alter much among AKI stages. Notably, the incidences of MAKE30 and one-year adverse outcomes in patients with the lowest AKI stage and severe AKD (AKD stage 2–3) were nearly two times higher than those in patients with the most severe AKI and AKD stage 0 (MAKE30:20.50% vs 10.44%; one-year adverse outcomes: 33.33% vs 14.84%). Multivariable logistic regression analysis stratified by AKI stage showed that AKD stage 2–3 remained significantly higher risks in developing MAKE30 and one-year adverse outcomes among each stratum of AKI; however, AKD stage 1 almost conferred no extra risk in MAKE30 and one-year adverse outcomes than AKD stage 0 (Table 4).

**Table 3:** Multivariable regression analysis of AKD stage on 30-day and one-year adverse outcomes.

Outcomes	AKD stage 1 OR/HR (95%CI)		AKD stage 2–3 OR/HR (95%CI)	
	Unadjusted^a^	Adjusted ^a^	Unadjusted^a^	Adjusted^a^
**30-day adverse outcomes**				
PRD^b^	7.37 (4.22–12.87)	6.13 (3.46–10.86)	160.86 (99.62–259.76)	143.33 (87.62–234.47)
New receipt of RRT^b^	6.52 (1.62–26.18)	4.02 (0.98–16.47)	39.42 (12.30–126.32)	18.86 (5.74–62.01)
Mortality^c^	1.88 (1.28–2.76)	1.47 (0.99–2.18)	4.27 (3.22–5.66)	2.52 (1.86–3.42)
MAKE 30^b^	2.97 (2.14–4.11)	2.36 (1.66–3.36)	34.70 (26.62–45.24)	31.35 (23.42–41.98)
**One-year adverse outcomes**				
Chronic dialysis^b^	0.82 (0.09–7.33)	0.41 (0.04–4.11)	10.42 (3.56–30.48)	9.85 (2.85–34.10)
Mortality^c^	1.44 (1.09–1.90)	1.24 (0.93–1.65)	2.94 (2.40–3.59)	2.08 (1.67–2.59)
Chronic dialysis and mortality^b^	1.45 (1.08–1.96)	1.21 (0.87–1.69)	3.56 (2.85–4.45)	2.68 (2.07–3.48)

**Notes.**

AKD acute kidney disease OR odds ratio HR hazard ratio PRD persistent renal dysfunction RRT renal replacement therapy MAKE 30 Major Adverse Kidney Events within 30 days

a AKD stage 0 was considered as the reference group.

b Multivariable logistic regression analysis was performed.

c Multivariable Cox regression analysis was performed.

**Table 4:** Unadjusted and adjusted odds ratio (95% confidence interval) of MAKE 30 and one-year adverse outcomes associated with AKD stage stratified by AKI stage.

	AKI stage 1	AKI stage 2	AKI stage 3
**MAKE30**			
**Unadjusted**			
AKD stage 0	1.00 (reference)	1.00 (reference)	1.00 (reference)
AKD stage 1	1.82 (0.83–4.03)	1.34 (0.73–2.43)	3.14 (1.76–5.58)
AKD stage 2–3	5.53 (2.38–12.87)	14.09 (8.84–22.45)	30.88 (18.40–51.85)
**Adjusted**			
AKD stage 0	1.00 (reference)	1.00 (reference)	1.00 (reference)
AKD stage 1	1.61 (0.66–3.94)	1.25 (0.65–2.39)	2.69 (1.46–4.97)
AKD stage 2–3	4.28 (1.39–13.16)	16.34 (9.68–27.59)	31.15 (17.98–53.96)
**One-year adverse outcomes**			
**Unadjusted**			
AKD stage 0	1.00 (reference)	1.00 (reference)	1.00 (reference)
AKD stage 1	1.78 (1.03–3.11)	1.21 (0.72–2.03)	1.13 (0.64–1.98)
AKD stage 2–3	4.16 (2.07–8.39)	2.71 (1.75–4.21)	2.94 (1.88–4.59)
**Adjusted**			
AKD stage 0	1.00 (reference)	1.00 (reference)	1.00 (reference)
AKD stage 1	1.54 (0.83–2.83)	1.13 (0.62–2.03)	0.95 (0.51–1.78)
AKD stage 2–3	3.14 (1.34–7.33)	2.28 (1.38–3.77)	2.27 (1.39–3.73)

**Notes.**

MAKE30, Major Adverse Kidney Events within 30 days; AKD, acute kidney disease; AKI, acute kidney injury.

Multivariable logistic regression analysis was performed with adjustment for age, sex, hypertension, diabetes mellitus, cardiac infarction, congestive heart failure, chronic liver disease, chronic kidney disease, cerebrovascular disease, cancer, sepsis, organ failure, Charlson comorbidity index, anemia, proteinuria, hyperuricemia, hypoalbuminemia, cardiovascular surgery and mechanical ventilation.

### Prediction of AKD stage 2–3

**Figure 4:**
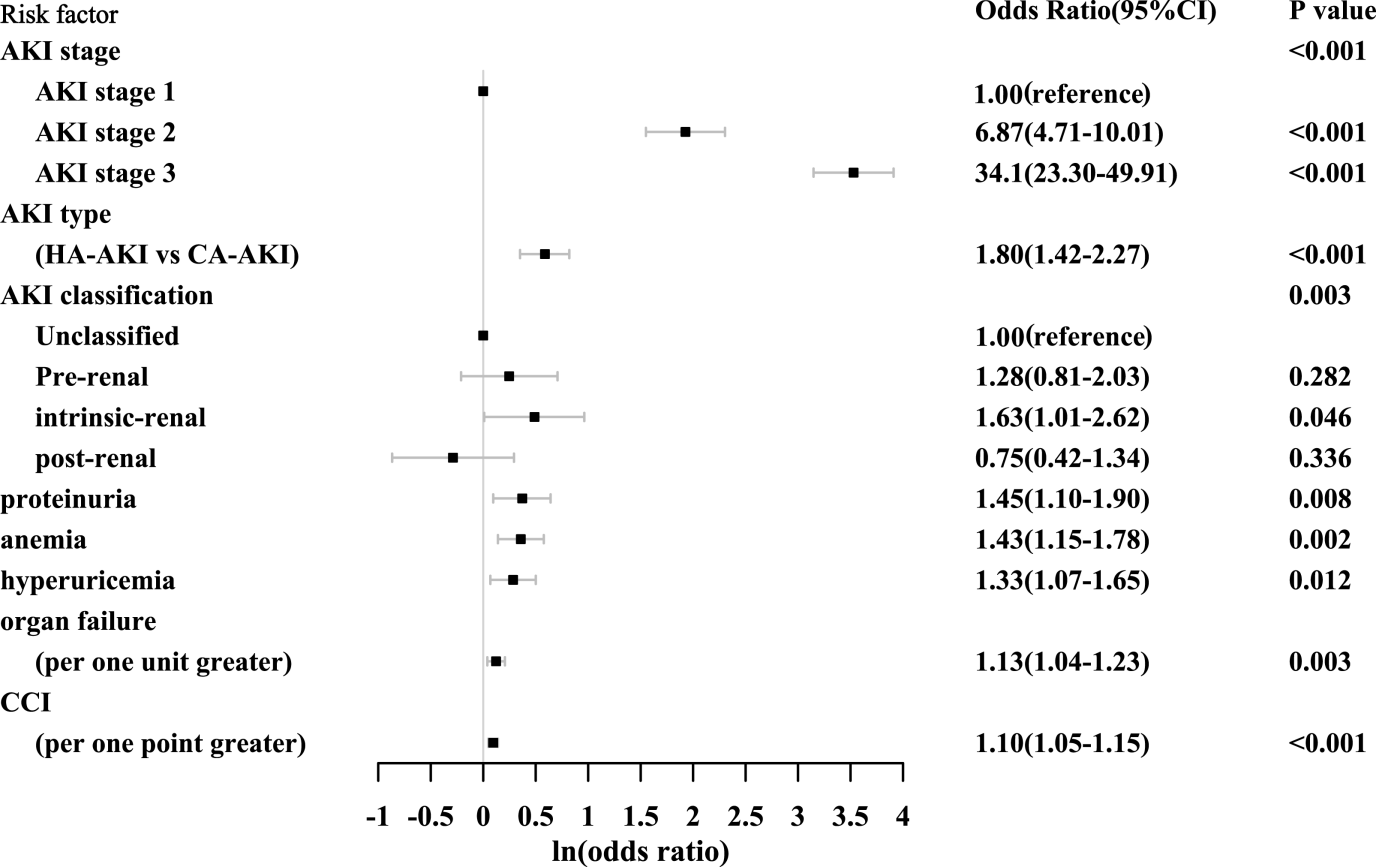
Risk factors for the development of AKD stage 2–3. AKD, acute kidney disease; CI, confidence interval; AKI, acute kidney injury; HA-AKI, hospital-acquired acute kidney injury; CA-AKI, community-acquired acute kidney injury; CCI, Charlson comorbidity index.

Multivariable logistic regression analysis showed that AKI stage, AKI type, AKI classification, proteinuria, anemia, hyperuricemia, organ failure as well as CCI were independent risk factors for the development of AKD stage 2–3 (Fig. 4). AKI stage was a major risk factor for AKD stage 2–3 with the highest odds ratio and predicted it in graded manner as the odds ratio got higher with the increase of AKI stage. This 8-variable model showed good discrimination and calibration in predicting AKD stage 2–3 with the AUROC being 0.85 (95% CI [0.83−0.87]) and the *P* value of the Hosmer and Lemeshow test being 0.78.

### Sensitivity analysis

The association between AKD stage and adverse outcomes in 30 days and one year was further examined in critically ill and non-critically ill patients. The results were not significantly changed as AKD stage 2–3 was similarly associated with increased risk of developing adverse outcomes in 30 days and one year both in the critically ill and non-critically ill, while the association of AKD stage 1 and one-year adverse outcomes was not seen in critically ill patients or non-critically ill patients (Tables S8, S9).

## Discussion

In our multi-center retrospective study, we found that AKD was commonly seen among hospitalized AKI patients as 45.4% of AKI patients would progress into AKD. AKD acted as an important intermediate stage between AKI and adverse outcomes in 30 days and one year. AKD stage 2–3 was associated with increased risk of 30-day and one-year adverse outcomes independent from AKI stage and the association did not alter much in critically ill and non-critically ill AKI patients.

Few studies so far have targeted on patients with AKD after AKI. The concept of AKD was first proposed by the KDIGO AKI workgroup in 2012, which was defined as any condition that impacts kidney function or structure lasting <3 months including AKI Kidney Disease: Improving Global Outcomes KDIGO, 2012. But in 2017, the ADQI workgroup proposed a new definition of AKD aiming to define the course of disease after AKI among patients in whom the renal pathophysiologic processes are ongoing. This new definition separates AKI and AKD as they reflect the severity of patient’ renal function injury within the first 6 days and 7-90 days after an AKI initiating event respectively, which is able to show the dynamic nature of renal function and natural course of the disease. Most studies on AKD so far took the definition by 2012 KDIGO AKI workgroup and targeted on AKD patients with or without AKI (Fujii et al., 2014; Hsu et al., 2020; James et al., 2019; Mima et al., 2019; Patimarattananan et al., 2020). The studies found that AKD patients not meeting the AKI criteria were associated with higher risks of developing new CKD, end stage kidney disease and death compared with patients without kidney injury (Fujii et al., 2014; Hsu et al., 2020; James et al., 2019). Researches adopting the 2017 ADQI workgroup definition and focusing on AKD patients after AKI have been scarce (Kofman et al., 2019; Matsuura et al., 2020; Peerapornratana et al., 2020). Kofman’s study (Kofman et al., 2019) enrolled 225 consecutive ST elevation myocardial infarction patients with AKI after percutaneous coronary intervention, and found that AKD was developed in 36% of the patients and associated with higher 90-day and long-term mortality. Other studies showed that AKD occurred in 47.1% of patients with AKI after cardiac surgery (Matsuura et al., 2020) and 26.9% of patients with septic shock and stage 2 or 3 AKI (Peerapornratana et al., 2020). But all these studies only recruited patients from particular clinical settings and had relatively small samples. And none of these studies included both renal dysfunction and hard clinical outcomes in the endpoints. Our study was the largest study so far to investigate the incidence and outcomes of patients with AKD after AKI. We enrolled all AKI patients admitted to three hospitals which can reduce the selection bias and make the population more representative. We employed MAKE30 which included two renal-specific events and mortality as our primary endpoints, which contained more clinical significance and can capture a greater percentage of patients with a meaningful poor outcome. The absence of MAKE30 is also an assessment of renal disability-free survival as well as an important goal for all patients and medical staff (Billings & Shaw, 2014).

Our study confirmed the important clinical significance of AKD. AKD is commonly seen in hospitalized AKI patients and represents a critical intermediate stage between AKI and adverse short- and long-term outcomes. AKD occurred in 45.4% of our study population which was consistent with previous finding (Matsuura et al., 2020). 86.5% of patients who developed MAKE30 and 64.8% of patients who developed chronic dialysis and mortality in one year were from AKD group. Multivariable regression analysis also indicated that AKD, especially AKD stage 2–3, was a strong predictor for the occurrence of MAKE30. AKD stage 2–3 carried more than 30 times the risk of developing MAKE30 and still showed 2.7 times higher risk in developing chronic dialysis and mortality in one year than non-AKD.

As studies have consistently shown that an episode of AKI was associated with increased risks for requirement of renal replacement therapy, subsequent CKD and mortality (Bucaloiu et al., 2012; Cheng et al., 2020; Hoste et al., 2018; Linder et al., 2014), whether the association between AKD and adverse outcomes in 30 days and one year was resulted from the impact of AKI stage was unclear. Therefore, we stratified the patients by both AKI stage and AKD stage. We found that the differences in the incidences of one-year adverse outcomes among AKI stages almost disappeared when patients were stratified by AKD stage. But when stratified by AKI stage, the incidences of one-year adverse outcomes kept increasing as AKD stage went higher, especially when AKD stage rose from stage 1 to stage 2–3. Notably, the incidences of MAKE30 or one-year adverse outcomes in patients with mild AKI and severe AKD (AKD stage 2–3) were nearly twice as high as those in patients with severe AKI without AKD. Moreover, AKD stage 2–3 was consistently and independently associated with increased risks of MAKE30 and one-year adverse outcomes in the adjusted multivariable regression models when controlled for AKI stage. Our findings extended the work on AKI and showed that AKD stage 2–3 could provide extra prognostic information over AKI stage which can help improve identification of patients with increased risk of adverse outcomes and raise awareness of AKD in clinical practice.

The findings in the present study can promote understanding of the pathophysiological changes of AKD. Clinically, we found AKI stage conferred an incremental and major risk of progressing into AKD stage 2–3 in a graded manner. Our result was supported by a previous study which demonstrated that patients with AKI stage 1, 2 and 3 had 2.3, 9.4 and 22.9 times higher risks of developing AKD (defined as doubling of creatinine 2–4 weeks after cardiac surgery) (Mizuguchi et al., 2018). Intrinsic renal injury and proteinuria were also independent risk factors for AKD stage 2–3. Proteinuria is regarded as a sign of renal structural damage while intrinsic AKI is usually characterized by tubular epithelial cell death. The results indicated that the occurrence of AKD stage 2–3 is the reflection of relatively severe renal injury and underlying renal structural damage. Pathophysiologically, progression from AKI depends on the balance of adaptive and maladaptive repair and persistence in renal injury results directly or secondly from maladaptive repair process (Basile et al., 2016). Several pathophysiologic processes including a reduction in capillary density (Basile et al., 2001; Kramann, Tanaka & Humphreys, 2014) and an expansion of interstitial fibroblasts and myofibroblasts (Venkatachalam et al., 2010) as well as some signaling pathways (Shu et al., 2019; Tang et al., 2019) activated during maladaptive repair which would promote interstitial fibrosis and lead to the development and progression of CKD and adverse outcomes. Taken together, the occurrence of severe AKD is the combined results from renal structural injury and responsive repair while the severity of renal injury can be the trigger of maladaptive repair process. AKD represents the endogenous renal repair process and an important transition period connecting AKI and adverse outcomes. Therefore, balancing the adaptive and maladaptive repair can be the target in preventing AKD occurrence and improving prognosis of AKI patients. Further studies are still needed to elucidate the mechanism of AKD to help find out possible intervening measures in an attempt to stop renal progression and facilitate recovery.

Our study also has some limitations. First, the possibility of misclassification in identifying exposures and associated variables in retrospective analysis along with unmeasured variables could potentially confound the relationship between AKD and outcomes. Second, the enrolled patients were restricted to academic hospitals, which might affect the general representativeness of the study population. Further studies performed in non-academic hospitals and other countries are needed to validate our findings. Third, only SCr value was used to identify AKI patients as the urinary data was unavailable for most patients. The results may have differed if urine output was included in identifying AKI patients. Finally, the data from after discharge to the time of death or study endpoint like patients’ blood pressure control and nephrotoxic drugs use are unavailable, therefore the effects of other insults and treatment on patients’ outcomes are unknown.

## Conclusion

AKD is common among AKI patients. AKD stage 2–3 is independently associated with increased risks of 30-day and one-year adverse outcomes and adds additional prognostic information over AKI stage. Awareness of potential risks associated with AKD stage 2–3 may help improve outcomes through careful monitoring and timely intervention.

##  Supplemental Information

10.7717/peerj.11400/supp-1 Supplemental Information 1 Odds ratio of all adjusted variables for persistent renal dysfunction in 30 days AKD, acute kidney disease; CKD, chronic kidney disease; CCI, Charlson comorbidity index.
Chi-square for the whole model was 1347.08, *P* < 0.001.

10.7717/peerj.11400/supp-2 Supplemental Information 2 Odds ratio of all adjusted variables for new receipt of RRT in 30 days RRT, renal replacement therapy; AKD, acute kidney disease; CKD, chronic kidney disease; CCI, Charlson comorbidity index.
Chi-square for the whole model was 164.59, *P* < 0.001.

10.7717/peerj.11400/supp-3 Supplemental Information 3 Odds ratio of all adjusted variables for mortality in 30 days AKD, acute kidney disease; CKD, chronic kidney disease; CCI, Charlson comorbidity index.
Chi-square for the whole model was 518.11, *P* < 0.001.

10.7717/peerj.11400/supp-4 Supplemental Information 4 Odds ratio of all adjusted variables for Major Adverse Kidney Events within 30 days AKD, acute kidney disease; CKD, chronic kidney disease; CCI, Charlson comorbidity index.
Chi-square for the whole model was 1160.77, *P* < 0.001.

10.7717/peerj.11400/supp-5 Supplemental Information 5 Odds ratio of all adjusted variables for RRT in one year AKD, acute kidney disease; CKD, chronic kidney disease; CCI, Charlson comorbidity index.
Chi-square for the whole model was 101.11, *P* < 0.001.

10.7717/peerj.11400/supp-6 Supplemental Information 6 Odds ratio of all adjusted variables for mortality in one year AKD, acute kidney disease; CKD, chronic kidney disease; CCI, Charlson comorbidity index.
Chi-square for the whole model was 582.08, *P* < 0.001.

10.7717/peerj.11400/supp-7 Supplemental Information 7 Odds ratio of all adjusted variables for mortality and RRT in one year RRT, renal replacement therapy; AKD, acute kidney disease; CKD, chronic kidney disease; CCI, Charlson comorbidity index.
Chi-square for the whole model was 448.03, *P* < 0.001.

10.7717/peerj.11400/supp-8 Supplemental Information 8 Outcomes of critically ill and non-critically ill patients stratified by AKD stages AKD, acute kidney disease; AKI, acute kidney injury; PRD, persistent renal dysfunction; RRT, renal replacement therapy.
^∗^ *P* < 0.05 compared with AKD stage 0; ^#^ *P* < 0.05 compared with AKD stage 1; ^*a*^ Comparison was made among AKD stage 0, 1 and 2–3.

10.7717/peerj.11400/supp-9 Supplemental Information 9 Adjusted multivariable regression analysis of AKD stage on 30-day and one-year adverse outcomes in critically ill and non-critically ill patients AKD: acute kidney disease; PRD: persistent renal dysfunction; RRT, renal replacement therapy; MAKE 30, Major Adverse Kidney Events within 30 days.
^*a*^ AKD stage 0 was considered as the reference group in multivariable regression analysis. age, sex, hypertension, diabetes mellitus, cardiac infarction, congestive heart failure, chronic liver disease, chronic kidney disease, cerebrovascular disease, cancer, sepsis, organ failure, Charlson comorbidity index, anemia, proteinuria, hyperuricemia, hypoalbuminemia, cardiovascular surgery and mechanical ventilation were added to the adjusted multivariable regression model.
^*b*^ AKD stage 0 was considered as the reference group in multivariable regression analysis. Mechanical ventilation was not added into the multivariable regression models compared with that in the critically ill patients.
^*c*^ Multivariable logistic regression analysis was performed.
^*d*^ Multivariable Cox regression analysis was performed.
^*e*^ Data were not available due to the small number of endpoints events.

10.7717/peerj.11400/supp-10 Supplemental Information 10 Raw data

10.7717/peerj.11400/supp-11 Supplemental Information 11 Explanation for categorical data in raw data

## Additional Information and Declarations

### Data Availability

The following information was supplied regarding data availability: The raw data are available in the Supplementary File.
