# Supplementary material for: Acute kidney disease in hospitalized acute kidney injury patients
Source: PeerJ. 2021 May 24;9:e11400. doi: 10.7717/peerj.11400 (PMC8158174; doi:10.7717/peerj.11400)
Supplement: Supplemental Information 1 — AKD, acute kidney disease; CKD, chronic kidney disease; CCI, Charlson comorbidity index. Chi-square for the whole model was 1347.08, P < 0.001. [file peerj-09-11400-s001.docx]

Supplemental Table 1. Odds ratio of all adjusted variables for persistent renal dysfunction in 30 days.

| Variables | Odds Ratio | 95% Confidence Interval | P value |
| --- | --- | --- | --- |
| AKD stage |  |  | <0.001 |
| stage 0 | 1.00 | reference |  |
| Stage 1 | 6.13 | (3.46-10.86) | <0.001 |
| Stage 2-3 | 143.33 | (87.62-234.47) | <0.001 |
| Age (≥65 vs < 65 years) | 1.32 | (0.92-1.91) | 0.13 |
| Sex (Male vs female) | 1.19 | (0.88-1.60) | 0.26 |
| Hypertension | 0.92 | (0.67-1.27) | 0.61 |
| Diabetes | 1.08 | (0.75-1.56) | 0.70 |
| Myocardial infarction | 1.44 | (0.73-2.84) | 0.29 |
| Congestive heart failure | 1.35 | (0.90-2.03) | 0.15 |
| Chronic liver disease | 0.91 | (0.65-1.28) | 0.60 |
| Cerebrovascular disease | 0.81 | (0.51-1.29) | 0.38 |
| CKD | 0.66 | (0.34-1.28) | 0.21 |
| Cancer | 1.12 | (0.77-1.63) | 0.56 |
| Sepsis | 0.66 | (0.42-1.03) | 0.07 |
| Organ failure (≥2 vs < 2) | 1.94 | (1.40-2.68) | <0.001 |
| CCI (≥2 vs <2 point) | 1.04 | (0.70-1.54) | 0.84 |
| Anemia | 1.26 | (0.93-1.71) | 0.13 |
| Proteinuria | 1.07 | (0.75-1.53) | 0.71 |
| Hyperuricemia | 1.23 | (0.92-1.64) | 0.16 |
| Hypoalbuminemia | 0.89 | (0.65-1.22) | 0.46 |
| Cardiovascular Surgery | 0.63 | (0.34-1.16) | 0.14 |
| Mechanical Ventilation | 1.03 | (0.70-1.52) | 0.87 |

AKD, acute kidney disease; CKD, chronic kidney disease; CCI, Charlson comorbidity index.

Chi-square for the whole model was 1347.08, P < 0.001.
