# Supplementary material for: Acute kidney disease in hospitalized acute kidney injury patients
Source: PeerJ. 2021 May 24;9:e11400. doi: 10.7717/peerj.11400 (PMC8158174; doi:10.7717/peerj.11400)
Supplement: Supplemental Information 2 — RRT, renal replacement therapy; AKD, acute kidney disease; CKD, chronic kidney disease; CCI, Charlson comorbidity index. Chi-square for the whole model was 164.59, P < 0.001. [file peerj-09-11400-s002.docx]

Supplemental Table 2. Odds ratio of all adjusted variables for new receipt of RRT in 30 days.

| Variables | Odds Ratio | 95% Confidence Interval | P value |
| --- | --- | --- | --- |
| AKD stage |  |  | <0.001 |
| stage 0 | 1.00 | reference |  |
| Stage 1 | 4.02 | (0.98-16.47) | 0.05 |
| Stage 2-3 | 18.86 | (5.74-62.01) | <0.001 |
| Age (≥65 vs < 65 years) | 0.87 | (0.43-1.75) | 0.69 |
| Sex (Male vs female) | 0.99 | (0.56-1.78) | 0.98 |
| Hypertension | 1.51 | (0.82-2.78) | 0.19 |
| Diabetes | 0.99 | (0.50-1.96) | 0.97 |
| Myocardial infarction | 0.67 | (0.14-3.12) | 0.61 |
| Congestive heart failure | 0.82 | (0.37-1.83) | 0.63 |
| Chronic liver disease | 1.29 | (0.69-2.40) | 0.43 |
| Cerebrovascular disease | 0.66 | (0.25-1.73) | 0.40 |
| CKD | 0.73 | (0.19-2.80) | 0.64 |
| Cancer | 1.27 | (0.61-2.63) | 0.52 |
| Sepsis | 1.05 | (0.49-2.27) | 0.90 |
| Organ failure (≥2 vs < 2) | 2.75 | (1.47-5.14) | 0.002 |
| CCI (≥2 vs <2 point) | 0.96 | (0.47-2.00) | 0.92 |
| Anemia | 2.33 | (1.31-4.14) | 0.004 |
| Proteinuria | 3.96 | (2.20-7.12) | <0.001 |
| Hyperuricemia | 1.13 | (0.64-1.97) | 0.67 |
| Hypoalbuminemia | 1.96 | (1.11-3.47) | 0.02 |
| Mechanical Ventilation | 1.05 | (0.53-2.07) | 0.90 |

RRT, renal replacement therapy; AKD, acute kidney disease; CKD, chronic kidney disease; CCI, Charlson comorbidity index.

Chi-square for the whole model was 164.59, P < 0.001.
