# Supplementary material for: Acute kidney disease in hospitalized acute kidney injury patients
Source: PeerJ. 2021 May 24;9:e11400. doi: 10.7717/peerj.11400 (PMC8158174; doi:10.7717/peerj.11400)
Supplement: Supplemental Information 3 — AKD, acute kidney disease; CKD, chronic kidney disease; CCI, Charlson comorbidity index. Chi-square for the whole model was 518.11, P < 0.001. [file peerj-09-11400-s003.docx]

Supplemental Table 3. Odds ratio of all adjusted variables for mortality in 30 days

| Variables | Odds Ratio | 95% Confidence Interval | P value |
| --- | --- | --- | --- |
| AKD stage |  |  | <0.001 |
| stage 0 | 1.00 | reference |  |
| Stage 1 | 1.47 | (0.99-2.18) | 0.06 |
| Stage 2-3 | 2.52 | (1.86-3.42) | <0.001 |
| Age (≥65 vs < 65 years) | 1.08 | (0.79-1.46) | 0.64 |
| Sex (Male vs female) | 0.93 | (0.70-1.22) | 0.57 |
| Hypertension | 0.95 | (0.72-1.25) | 0.71 |
| Diabetes | 0.87 | (0.64-1.19) | 0.37 |
| Myocardial infarction | 1.06 | (0.65-1.72) | 0.83 |
| Congestive heart failure | 1.15 | (0.83-1.61) | 0.41 |
| Chronic liver disease | 1.03 | (0.77-1.39) | 0.82 |
| Cerebrovascular disease | 1.25 | (0.89-1.74) | 0.20 |
| CKD | 1.08 | (0.60-1.94) | 0.79 |
| Cancer | 1.64 | (1.21-2.24) | <0.001 |
| Sepsis | 1.10 | (0.80-1.51) | 0.55 |
| Organ failure (≥2 vs < 2) | 2.00 | (1.49-2.69) | <0.001 |
| CCI (≥2 vs <2 point) | 1.81 | (1.26-2.62) | <0.001 |
| Anemia | 1.10 | (0.85-1.43) | 0.46 |
| Proteinuria | 0.89 | (0.63-1.25) | 0.50 |
| Hyperuricemia | 1.22 | (0.94-1.58) | 0.13 |
| Hypoalbuminemia | 1.10 | (0.83-1.44) | 0.51 |
| Cardiovascular Surgery | 0.78 | (0.47-1.29) | 0.33 |
| Mechanical Ventilation | 4.76 | (3.50-6.46) | <0.001 |

AKD, acute kidney disease; CKD, chronic kidney disease; CCI, Charlson comorbidity index.

Chi-square for the whole model was 518.11, P < 0.001.
