# Supplementary material for: Acute kidney disease in hospitalized acute kidney injury patients
Source: PeerJ. 2021 May 24;9:e11400. doi: 10.7717/peerj.11400 (PMC8158174; doi:10.7717/peerj.11400)
Supplement: Supplemental Information 4 — AKD, acute kidney disease; CKD, chronic kidney disease; CCI, Charlson comorbidity index. Chi-square for the whole model was 1160.77, P < 0.001. [file peerj-09-11400-s004.docx]

Supplemental Table 4. Odds ratio of all adjusted variables for Major Adverse Kidney Events within 30 days.

| Variables | Odds Ratio | 95% Confidence Interval | P value |
| --- | --- | --- | --- |
| AKD stage |  |  | <0.001 |
| stage 0 | 1.00 | reference |  |
| Stage 1 | 2.36 | (1.66-3.36) | <0.001 |
| Stage 2-3 | 31.35 | (23.42-41.98) | <0.001 |
| Age (≥65 vs < 65 years) | 1.17 | (0.86-1.60) | 0.32 |
| Sex (Male vs female) | 1.17 | (0.91-1.60) | 0.23 |
| Hypertension | 0.93 | (0.71-1.23) | 0.63 |
| Diabetes | 0.88 | (0.64-1.21) | 0.44 |
| Myocardial infarction | 1.37 | (0.75-2.50) | 0.31 |
| Congestive heart failure | 1.41 | (0.99-2.01) | 0.06 |
| Chronic liver disease | 0.82 | (0.61-1.10) | 0.19 |
| Cerebrovascular disease | 0.89 | (0.60-1.32) | 0.57 |
| CKD | 0.89 | (0.50-1.59) | 0.70 |
| Cancer | 1.26 | (0.91-1.73) | 0.16 |
| Sepsis | 0.90 | (0.60-1.34) | 0.59 |
| Organ failure (≥2 vs < 2) | 2.08 | (1.58-2.75) | <0.001 |
| CCI (≥2 vs <2 point) | 1.40 | (1.00-1.95) | 0.05 |
| Anemia | 1.42 | (1.09-1.84) | 0.01 |
| Proteinuria | 1.10 | (0.80-1.52) | 0.54 |
| Hyperuricemia | 1.21 | (0.95-1.56) | 0.13 |
| Hypoalbuminemia | 0.98 | (0.74-1.29) | 0.87 |
| Cardiovascular Surgery | 0.40 | (0.23-0.69) | <0.001 |
| Mechanical Ventilation | 2.87 | (2.07-3.98) | <0.001 |

AKD, acute kidney disease; CKD, chronic kidney disease; CCI, Charlson comorbidity index.

Chi-square for the whole model was 1160.77, P < 0.001.
