# Supplementary material for: Acute kidney disease in hospitalized acute kidney injury patients
Source: PeerJ. 2021 May 24;9:e11400. doi: 10.7717/peerj.11400 (PMC8158174; doi:10.7717/peerj.11400)
Supplement: Supplemental Information 5 — AKD, acute kidney disease; CKD, chronic kidney disease; CCI, Charlson comorbidity index. Chi-square for the whole model was 101.11, P < 0.001. [file peerj-09-11400-s005.docx]

Supplemental Table 5. Odds ratio of all adjusted variables for RRT in one year.

| Variables | Odds Ratio | 95% Confidence Interval | P value |
| --- | --- | --- | --- |
| AKD stage |  |  | <0.001 |
| AKD stage 0 | 1.00 | reference | 0.00 |
| AKD stage 1 | 0.41 | (0.04-4.11) | 0.45 |
| AKD stage 2-3 | 9.85 | (2.85-34.10) | 0.00 |
| Age (≥65 vs < 65 years) | 0.78 | (0.27-2.26) | 0.65 |
| Sex (Male vs female) | 0.79 | (0.30-2.06) | 0.62 |
| Hypertension | 3.63 | (1.33-9.89) | 0.01 |
| Diabetes | 1.79 | (0.68-4.73) | 0.24 |
| Myocardial infarction | 0.51 | (0.05-5.35) | 0.57 |
| Congestive heart failure | 0.52 | (0.14-1.86) | 0.31 |
| Chronic liver disease | 1.09 | (0.38-3.14) | 0.87 |
| Cerebrovascular disease | 0.40 | (0.08-1.97) | 0.26 |
| CKD | 8.90 | (2.69-29.44) | 0.00 |
| Cancer | 0.15 | (0.02-1.31) | 0.09 |
| Organ failure (≥2 vs < 2) | 0.35 | (0.12-1.05) | 0.06 |
| CCI (≥2 vs <2 point) | 2.15 | (0.57-8.05) | 0.26 |
| Anemia | 0.92 | (0.35-2.45) | 0.87 |
| Proteinuria | 2.35 | (0.92-5.98) | 0.07 |
| Hyperuricemia | 0.46 | (0.18-1.17) | 0.10 |
| Hypoalbuminemia | 4.62 | (1.69-12.68) | 0.00 |

AKD, acute kidney disease; CKD, chronic kidney disease; CCI, Charlson comorbidity index.

Chi-square for the whole model was 101.11, P < 0.001.
