# Supplementary material for: Acute kidney disease in hospitalized acute kidney injury patients
Source: PeerJ. 2021 May 24;9:e11400. doi: 10.7717/peerj.11400 (PMC8158174; doi:10.7717/peerj.11400)
Supplement: Supplemental Information 6 — AKD, acute kidney disease; CKD, chronic kidney disease; CCI, Charlson comorbidity index. Chi-square for the whole model was 582.08, P < 0.001. [file peerj-09-11400-s006.docx]

Supplemental Table 6. Odds ratio of all adjusted variables for mortality in one year.

| Variables | Odds Ratio | 95% Confidence Interval | P value |
| --- | --- | --- | --- |
| AKD stage |  |  | <0.001 |
| stage 0 | 1.00 | reference |  |
| Stage 1 | 1.24 | (0.93-1.65) | 0.14 |
| Stage 2-3 | 2.08 | (1.67-2.59) | <0.001 |
| Age (≥65 vs < 65 years) | 1.19 | (0.95-1.49) | 0.13 |
| Sex (Male vs female) | 0.78 | (0.63-0.96) | 0.02 |
| Hypertension | 0.90 | (0.73-1.12) | 0.35 |
| Diabetes | 1.00 | (0.79-1.26) | 0.99 |
| Myocardial infarction | 0.95 | (0.63-1.43) | 0.81 |
| Congestive heart failure | 1.50 | (1.16-1.94) | <0.001 |
| Chronic liver disease | 1.02 | (0.82-1.27) | 0.85 |
| Cerebrovascular disease | 1.24 | (0.96-1.61) | 0.11 |
| CKD | 0.83 | (0.51-1.34) | 0.44 |
| Cancer | 2.19 | (1.75-2.75) | 0.00 |
| Sepsis | 1.26 | (0.98-1.63) | 0.08 |
| Organ failure (≥2 vs < 2) | 1.63 | (1.31-2.03) | <0.001 |
| CCI (≥2 vs <2 point) | 1.75 | (1.33-2.30) | <0.001 |
| Anemia | 1.12 | (0.92-1.36) | 0.27 |
| Proteinuria | 0.94 | (0.73-1.23) | 0.66 |
| Hyperuricemia | 1.16 | (0.96-1.41) | 0.13 |
| Hypoalbuminemia | 1.10 | (0.89-1.35) | 0.37 |
| Cardiovascular Surgery | 0.74 | (0.49-1.11) | 0.15 |
| Mechanical Ventilation | 3.07 | (2.44-3.85) | <0.001 |

AKD, acute kidney disease; CKD, chronic kidney disease; CCI, Charlson comorbidity index.

Chi-square for the whole model was 582.08, P < 0.001.
