# Supplementary material for: Acute kidney disease in hospitalized acute kidney injury patients
Source: PeerJ. 2021 May 24;9:e11400. doi: 10.7717/peerj.11400 (PMC8158174; doi:10.7717/peerj.11400)
Supplement: Supplemental Information 7 — RRT, renal replacement therapy; AKD, acute kidney disease; CKD, chronic kidney disease; CCI, Charlson comorbidity index. Chi-square for the whole model was 448.03, P < 0.001. [file peerj-09-11400-s007.docx]

Supplemental Table 7. Odds ratio of all adjusted variables for mortality and RRT in one year.

| Variables | Odds Ratio | 95% Confidence Interval | P value |
| --- | --- | --- | --- |
| AKD stage |  |  | <0.001 |
| stage 0 | 1.00 | reference |  |
| Stage 1 | 1.21 | (0.87-1.69) | 0.26 |
| Stage 2-3 | 2.68 | (2.07-3.48) | <0.001 |
| Age (≥65 vs < 65 years) | 1.18 | (0.90-1.55) | 0.24 |
| Sex (Male vs female) | 0.73 | (0.57-0.93) | 0.01 |
| Hypertension | 0.99 | (0.77-1.29) | 0.96 |
| Diabetes | 1.10 | (0.83-1.45) | 0.53 |
| Myocardial infarction | 0.89 | (0.52-1.53) | 0.67 |
| Congestive heart failure | 1.68 | (1.22-2.31) | <0.001 |
| Chronic liver disease | 1.01 | (0.77-1.33) | 0.92 |
| Cerebrovascular disease | 1.23 | (0.88-1.73) | 0.22 |
| CKD | 1.17 | (0.69-1.96) | 0.56 |
| Cancer | 2.46 | (1.86-3.26) | <0.001 |
| Sepsis | 1.45 | (1.03-2.04) | 0.04 |
| Organ failure (≥2 vs < 2) | 1.59 | (1.23-2.06) | 0.00 |
| CCI (≥2 vs <2 point) | 1.97 | (1.43-2.71) | <0.001 |
| Anemia | 1.18 | (0.93-1.50) | 0.18 |
| Proteinuria | 1.15 | (0.84-1.57) | 0.39 |
| Hyperuricemia | 1.04 | (0.82-1.32) | 0.74 |
| Hypoalbuminemia | 1.25 | (0.97-1.61) | 0.09 |
| Cardiovascular Surgery | 0.65 | (0.40-1.07) | 0.09 |
| Mechanical Ventilation | 3.46 | (2.61-4.57) | <0.001 |

RRT, renal replacement therapy; AKD, acute kidney disease; CKD, chronic kidney disease; CCI, Charlson comorbidity index.

Chi-square for the whole model was 448.03, P < 0.001.
