# Supplementary material for: Acute kidney disease in hospitalized acute kidney injury patients
Source: PeerJ. 2021 May 24;9:e11400. doi: 10.7717/peerj.11400 (PMC8158174; doi:10.7717/peerj.11400)
Supplement: Supplemental Information 8 — AKD, acute kidney disease; AKI, acute kidney injury; PRD, persistent renal dysfunction; RRT, renal replacement therapy. ∗ P < 0.05 compared with AKD stage 0; # P < 0.05 compared with AKD stage 1; a Comparison was made among AKD stage 0, 1 and 2–3. [file peerj-09-11400-s008.docx]

Supplemental Table 8. Outcomes of critically ill and non-critically ill patients stratified by AKD stages.

|  | Critically ill patients (N = 821) | | | |  | Non-critically ill (N = 1735) | | | |
| --- | --- | --- | --- | --- | --- | --- | --- | --- | --- |
| Outcomes | AKD stage 0 N=437 n (%) | AKD stage 1 N=130 n (%) | AKD stage 2-3 N=254 n (%) | *P* value*^a^* | | AKD stage 0 N= 958 n (%) | AKD stage 1 N= 303 n (%) | AKD stage 2-3 N= 474 n (%) | *P* value*^a^* |
| Major Adverse Kidney Events within 30 days | | | | | | | | | |
| PRD | 1  (0.2%) | 9  (6.9%)^*^ | 180 (70.9%)^*#^ | < 0.001 | | 18  (1.9%) | 31 (10.2%)^*^ | 322 (67.9%)^*#^ | <0.001 |
| New RRT | 2  (0.5%) | 3  (2.3%) | 22 (8.7%)^*#^ | < 0.001 | | 1  (0.1%) | 3  (1.0%) | 35 (7.4%)^*#^ | <0.001 |
| Mortality | 40 (9.2%) | 25 (19.2%)^*^ | 83 (32.7%)^*#^ | < 0.001 | | 32  (3.3%) | 16  (5.3%) | 64 (13.5%)^*#^ | <0.001 |
| Total | 42 (9.6%) | 32 (24.6%)^*^ | 192 (75.6%)^*#^ | < 0.001 | | 50  (5.2%) | 43 (14.2%)^*^ | 325 (68.4%)^*#^ | <0.001 |
| One-year adverse outcomes | | | | | | | | | |
| Chronic dialysis | 0  (0.0%) | 0  (0.0%) | 2  (0.8%) | 0.107 | | 4  (0.4%) | 1  (0.3%) | 19 (4.0%)^*#^ | <0.001 |
| Mortality | 64 (14.6%) | 35 (26.9%)^*^ | 111 (43.7%)^*#^ | < 0.001 | | 103  (10.8%) | 37 (12.2%) | 111 (23.4%)^*#^ | <0.001 |
| Total | 64 (14.6%) | 35 (26.9%)^*^ | 113 (44.5%)^*#^ | < 0.001 | | 107  (11.2%) | 38 (12.5%) | 129 (27.2%)^*#^ | <0.001 |

AKD, acute kidney disease; AKI, acute kidney injury; PRD, persistent renal dysfunction; RRT, renal replacement therapy.

^*^ P < 0.05 compared with AKD stage 0;

^#^ P < 0.05 compared with AKD stage 1;

*^a^* Comparison was made among AKD stage 0, 1 and 2-3.
