# Supplementary material for: Acute kidney disease in hospitalized acute kidney injury patients
Source: PeerJ. 2021 May 24;9:e11400. doi: 10.7717/peerj.11400 (PMC8158174; doi:10.7717/peerj.11400)
Supplement: Supplemental Information 9 — AKD: acute kidney disease; PRD: persistent renal dysfunction; RRT, renal replacement therapy; MAKE 30, Major Adverse Kidney Events within 30 days. a AKD stage 0 was considered as the reference group in multivariable regression analysis. age, sex, hypertension, diabetes mellitus, cardiac infarction, congestive heart failure, chronic liver disease, chronic kidney disease, cerebrovascular disease, cancer, sepsis, organ failure, Charlson comorbidity index, anemia, proteinuria, hyperuricemia, hypoalbuminemia, cardiovascular surgery and mechanical ventilation were added to the adjusted multivariable regression model. b AKD stage 0 was considered as the reference group in multivariable regression analysis. Mechanical ventilation was not added into the multivariable regression models compared with that in the critically ill patients. c Multivariable logistic regression analysis was performed. d Multivariable Cox regression analysis was performed. e Data were not available due to the small number of endpoints events. [file peerj-09-11400-s009.docx]

Supplemental Table 9. Adjusted multivariable regression analysis of AKD stage on 30-day and one-year adverse outcomes in critically ill and non-critically ill patients.

|  | Critically ill*^a^* | |  | Non-critically ill*^b^* | |
| --- | --- | --- | --- | --- | --- |
| Outcomes | AKD  stage 1 | AKD  stage2-3 | | AKD  stage 1 | AKD  stage 2-3 |
| **30-day adverse outcomes** | | | | | |
| PRD*^c^* | 22.78  (2.78-186.36) | 911.01  (123.77-6705.49) | | 5.06  (2.73-9.36) | 111.63  (65.68-189.71) |
| New receipt of RRT*^c^* | 2.11  (0.31-14.27) | 8.67  (1.86-40.28) | | 6.77  (0.69-66.89) | 43.32  (5.76-325.94) |
| Mortality*^d^* | 1.50  (0.89-2.52) | 2.58  (1.71-3.88) | | 1.41  (0.76-2.60) | 3.06  (1.93-4.85) |
| MAKE 30*^c^* | 2.02  (1.15-3.59) | 23.94  (14.80-38.71) | | 2.50  (1.59-3.94) | 38.72  (26.61-56.33) |
| **One-year adverse outcomes** | | | | | |
| Chronic dialysis*^c^* | —*^e^* | —*^e^* | | 0.45  (0.045-4.46) | 12.21  (3.32-44.86) |
| Mortality*^d^* | 1.42  (0.92-2.18) | 2.46  (1.76-3.46) | | 1.15  (0.78-1.69) | 2.07  (1.54-2.78) |
| Chronic dialysis  and mortality*^c^* | 1.47  (0.86-2.49) | 3.31  (2.17-5.06) | | 1.08  (0.70-1.65) | 2.65  (1.90-3.69) |

AKD: acute kidney disease; PRD: persistent renal dysfunction; RRT, renal replacement therapy; MAKE 30, Major Adverse Kidney Events within 30 days.

*^a^* AKD stage 0 was considered as the reference group in multivariable regression analysis. age, sex, hypertension, diabetes mellitus, cardiac infarction, congestive heart failure, chronic liver disease, chronic kidney disease, cerebrovascular disease, cancer, sepsis, organ failure, Charlson comorbidity index, anemia, proteinuria, hyperuricemia, hypoalbuminemia, cardiovascular surgery and mechanical ventilation were added to the adjusted multivariable regression model.

*^b^* AKD stage 0 was considered as the reference group in multivariable regression analysis. Mechanical ventilation was not added into the multivariable regression models compared with that in the critically ill patients.

*^c^* Multivariable logistic regression analysis was performed.

*^d^* Multivariable Cox regression analysis was performed.

^e^ Data were not available due to the small number of endpoints events.
