# Supplementary material for: Acute kidney disease in hospitalized acute kidney injury patients
Source: PeerJ. 2021 May 24;9:e11400. doi: 10.7717/peerj.11400 (PMC8158174; doi:10.7717/peerj.11400)
Supplement: Supplemental Information 11 [file peerj-09-11400-s011.docx]

Explanation for categorical data in raw data

1. Column F: Age2category: age≤65 was classified as 1，age > 65 was classified as 2.
2. Column AH: AKI classification: 1: pre-renal AKI; 2: intrinsic-renal AKI; 3:post-renal AKI;

4: unclassified AKI

1. Column AJ: AKI type: 1: community-acquired AKI; 2: hospital-acquired AKI
2. Column AT: organ failure 2 category: number of organ failure < 2 was classified as 0, number of organ failure ≥ 2 was classified as 1.
3. Column BI: Charlson score 2 categories: Charlson score ≤ 2 was classified as 0, Charlson score > 2 was classified as 1.
